# Trigeminal Neuralgia-Divided but Not Classified

**DOI:** 10.5812/aapm.3761

**Published:** 2012-09-13

**Authors:** Erlick Abilio Coelho Pereira

**Affiliations:** 1 Department of Neurological Surgery, Oxford University Hospitals, Oxford, UK; 2 Nuffield Department of Surgical Sciences, Oxford University Hospitals, Oxford, UK

**Keywords:** Trigeminal Neuralgia, Pain

Dear Editor,

Trigeminal neuralgia (TN) is “a sudden, usually unilateral, severe, brief, stabbing, recurrent pain in the distribution of one or more branches of the fifth cranial nerve” according to the international association for the study of pain (1). It is a debilitating condition of clinical importance not just because recent British and Dutch epidemiological studies show it to be more common than previously thought, (2) affecting close to 30 per 100,000 people, but also because it is commonly misdiagnosed by dental and medical professionals.

Correct diagnosis enables treatment and anatomic localisation directs proper intervention. Medical measures include neuropathic pharmacotherapies such as carbamazepine, oxcarbazapine, lamotrigine, gabapentin, pregabalin, phenyotin and valproate. Neurosurgery ranges from percutaneous ablation by cryosurgery, radiofrequency thermocoagulative rhizolysis, balloon compression and glycerol injection to gamma knife radiosurgery and microvascular decompression of the trigeminal nerve, either open, or endoscope assisted through a ‘keyhole’. The variety of surgical treatments are targeted either at the Gasserian ganglion, distal to it or at the brainstem dorsal root entry zone of the trigeminal nerve (3).

Bangash (4) has performed a cross-sectional study of 100 patients presenting to a single Pakistani centre over one year to suggest that TN occurs twice as often in females and is twice as likely to be on the right side of the face. It affected the mandibular division of the nerve in the majority and seldom the ophthalmic division with concomitant maxillary and mandibular involvement seen in a small proportion of patients. Repeated diagnostic injection of local anaesthetic helped localise the pain.

The study may have benefitted from its author undertaking neurophysiologic tests to assess trigeminal reflexes and evoked potentials, and from a description of the treatments given and their outcomes. Furthermore, attempts could have been made to classify the presentations of TN by the degree to which the pain was episodic (type 1) or constant (type 2), whether prior trigeminal injury or deafferentation had occurred, and if multiple sclerosis, Herpes zoster or a comorbid somatisation disorder was present (5). This classification proposed by Burchiel attempts to characterise less ambiguously the symptoms of TN and is gaining popularity in studies of TN treatment outcomes. Nevertheless, Bangash provides a welcome addition to the epidemiological and symptomatological literature surrounding an important and painful problem.
